# Minimally invasive drainage in critically ill patients with severe necrotizing pancreatitis is associated with better outcomes: an observational study

**DOI:** 10.1186/s13054-018-2256-x

**Published:** 2018-11-22

**Authors:** Lucie Darrivere, Nathanael Lapidus, Nikias Colignon, Najim Chafai, Ulriikka Chaput, Franck Verdonk, François Paye, Thomas Lescot

**Affiliations:** 1 Department of Anesthesiology and Critical Care Medicine, Saint-Antoine Hospital, Assistance Publique-Hôpitaux de Paris, Paris, France; 2 Sorbonne University, INSERM, Institut Pierre Louis d’Epidémiologie et de Santé Publique IPLESP, Public Health Department, Saint-Antoine Hospital, Assistance Publique-Hôpitaux de Paris, Paris, France; 3 Radiology Department, Saint-Antoine Hospital, Assistance Publique-Hôpitaux de Paris, Paris, France; 4 Digestive Surgery Department, Saint-Antoine Hospital, Assistance Publique-Hôpitaux de Paris, Paris, France; 5 Endoscopy Department, Saint-Antoine Hospital, Assistance Publique-Hôpitaux de Paris, Paris, France; 6 Sorbonne University, Department of Anesthesiology and Critical Care Medicine, Saint-Antoine Hospital, Assistance Publique-Hôpitaux de Paris, Paris, France; 7 Sorbonne University, Digestive Surgery Department, Saint-Antoine Hospital, Assistance Publique-Hôpitaux de Paris, Paris, France

**Keywords:** Severe acute pancreatitis, Infected pancreatic necrosis, Step-up approach, Surgical pancreatic necrosectomy

## Abstract

**Background:**

Infected pancreatic necrosis, which occurs in about 40% of patients admitted for acute necrotizing pancreatitis, requires combined antibiotic therapy and local drainage. Since 2010, drainage by open surgical necrosectomy has been increasingly replaced by less invasive methods such as percutaneous radiological drainage, endoscopic necrosectomy, and laparoscopic surgery, which proved effective in small randomized controlled trials in highly selected patients. Few studies have evaluated minimally invasive drainage methods used under the conditions of everyday hospital practice. The aim of this study was to determine whether, compared with conventional open surgery, minimally invasive drainage was associated with improved outcomes of critically ill patients with infection complicating acute necrotizing pancreatitis.

**Methods:**

A single-center observational study was conducted in patients admitted to the intensive care unit for severe acute necrotizing pancreatitis to compare the characteristics, drainage techniques, and outcomes of the 62 patients managed between September 2006 and December 2010, chiefly with conventional open surgery, and of the 81 patients managed between January 2011 and August 2015 after the introduction of a minimally invasive drainage protocol.

**Results:**

Surgical necrosectomy was more common in the early period (74% versus 41%; *P* <0.001), and use of minimally invasive drainage increased between the early and late periods (19% and 52%, respectively; *P* <0.001). The numbers of ventilator-free days and catecholamine-free days by day 30 were higher during the later period. The proportions of patients discharged from intensive care within the first 30 days and from the hospital within the first 90 days were higher during the second period. Hospital mortality was not significantly different between the early and late periods (19% and 22%, respectively).

**Conclusion:**

In our study, the implementation of a minimally invasive drainage protocol in patients with infected pancreatic necrosis was associated with shorter times spent with organ dysfunction, in the intensive care unit, and in the hospital. Mortality was not significantly different. These results should be interpreted bearing in mind the limitations inherent in the before-after study design.

## Background

Acute pancreatitis is a common condition whose incidence has increased recently, being estimated in 2013 at 13 to 45 cases/100,000 population in the US [1]. Mortality ranges from 1% to 2% overall. However, pancreatic necrosis develops in 10% to 20% of patients and is associated with complications and a higher mortality rate of up to 30% [2]. Secondary infection of necrotic tissue is a further aggravating factor that is diagnosed in about 40% of patients and is associated with death [3–5]. Until recently, the standard treatment of proven or suspected infected pancreatic necrosis (IPN) was open surgical necrosectomy (OSN). This procedure triggers a strong inflammatory response that can lead to prolonged multiorgan failure and result in local complications such as bleeding and gastrointestinal fistula [6]. Several minimally invasive drainage (MID) methods were introduced recently. They include imaging-guided percutaneous catheter drainage, transluminal endoscopic necrosectomy through the stomach or duodenum, and retroperitoneal surgical drainage. The use of MID for the first-line local treatment of IPN has been suggested. In small randomized controlled trials, patient outcomes were better in the MID arms than in the OSN arms [7, 8]: first-line imaging-guided percutaneous drainage or endoscopic transgastric necrosectomy reduced the rate of a composite endpoint of major complication or death compared with OSN. However, in another randomized trial comparing an endoscopic step-up approach and a surgical step-up approach in 98 patients, neither major complications nor mortality differed between the two groups, although fistulas were less common and hospital stays shorter in the endoscopy arm [9]. However, although these studies were methodologically sound, they included highly selected patients, most of whom had no organ failures. Of 11 studies in 384 patients (with a single randomized trial) included in a systematic review, only four reported data on organ failures [10]. In clinical practice, however, organ failures are common in patients with IPN.

The objective of this study was to assess the outcomes of MID versus OSN in unselected patients with IPN admitted to the intensive care unit (ICU). We compared two groups of patients admitted before and after the implementation of an MID protocol in 2011 in our ICU.

## Methods

### Study design and population

We conducted a retrospective, before-after, single-center observational study from September 2006 to August 2015 at the surgical ICU of the Anaesthesiology and Critical Care Department of the Saint-Antoine University Hospital in Paris, France. Consecutive patients older than 18 years and admitted to the ICU for severe acute necrotizing pancreatitis were included. The study was approved by the French Data Protection Authority (*Commission National Informatique et Libertés*, #2152259) and the French Anaesthesiology and Critical Care Research Ethics Committee (CERAR, IRB #00010254-2018-019), which waived the need for individual informed consent in accordance with French law on retrospective studies of anonymized data.

Patients included in the study were treated in accordance with international guidelines adapted to our local resources and procedures. Exclusion criteria were postsurgical acute pancreatitis, acute pancreatitis as a secondary diagnosis, and missing clinical and laboratory data. IPN was suspected if a prolonged fever (>38.5 °C for >5 days) was combined with an elevated leukocyte count or a new organ failure or gas visible within the pancreatic collection or a combination of these factors. A definitive diagnosis of IPN was defined as a positive microbiological result of a sample collected by aspiration under ultrasound or computed tomography (CT) guidance. In January 2011, the drainage protocol for patients with IPN was changed in our ICU: MID instead of OSN was used as the first-line drainage technique. To assess the possible impact of this change, we compared patient characteristics, drainage techniques, and outcomes between the groups included before and after the change.

Indications for drainage during both periods were suspected or proven necrosis infection, abdominal compartment syndrome, and local mechanical complications. During the earlier period, OSN was the treatment of choice in patients with positive microbiological results of samples collected by aspiration under ultrasound or CT guidance. Surgery was usually performed through a bilateral subcostal incision and consisted of removal of the necrotic tissue followed by continuous irrigation and drainage [11]. Starting in January 2011, MID methods were routinely considered for first-line use in patients with proven IPN. Two MID methods were used: CT-guided percutaneous drainage and endoscopic transgastric necrosectomy. CT-guided percutaneous drainage was often performed first. If this was unsuccessful, further drainage modalities were discussed during a staff meeting on the basis of the location of the necrotic tissue, and preference was given to MID methods.

### Data collection

For this retrospective study, the following data were collected from the electronic files and patient charts: patient characteristics (gender, weight, height, body mass index, Sequential Organ Failure Assessment [SOFA] score, location of patients just before admission to our ICU, and arterial lactate at admission), pancreatitis characteristics (etiology, Balthazar score, percentage of necrotised parenchyma, and whether necrosis infection developed), treatment strategy in the event of IPN (OSN or MID, with the MID method or methods), and outcomes (days on mechanical ventilation [MV], renal replacement therapy [RRT], and catecholamines; 90-day hospital mortality; and ICU and hospital lengths of stay).

### Statistical analysis

Patient demographic characteristics were described as median [interquartile range, or IQR] for quantitative variables and as number (percentage) for categorical variables. Comparisons of these characteristics between the two periods relied on the Mann–Whitney–Wilcoxon test for quantitative variables (including days without MV, RRT, and catecholamines over the first 30 ICU days) and on Fisher’s exact test for categorical variables. MV-free, RRT-free, and catecholamine-free days were evaluated only in patients admitted directly to our hospital, as the relevant data were missing for the other patients. The proportion of necrotised pancreatic parenchyma was compared between the periods by using the chi-squared test for trend in proportions. To compare day-30 survival between groups, we used proportional odds Cox models, expressing the results as hazard ratios (HRs) and 95% confidence intervals (CIs) with the earlier period as the reference. Cumulative mortality was estimated with the Kaplan–Meier estimator. Proportional odds models were also built to compare ICU discharge by day 30 and hospital discharge by day 90, and death was a competing risk. Cumulative incidences of ICU or hospital discharge were estimated with Gray estimators [12]. All survival models were adjusted on age and SOFA at admission. All tests were two-tailed and *P* values lower than 0.05 were considered significant. Statistical analyses were performed by using R software version 3.3 (R Foundation for Statistical Computing, Vienna, Austria).

## Results

### Patients

**Fig. 1:**
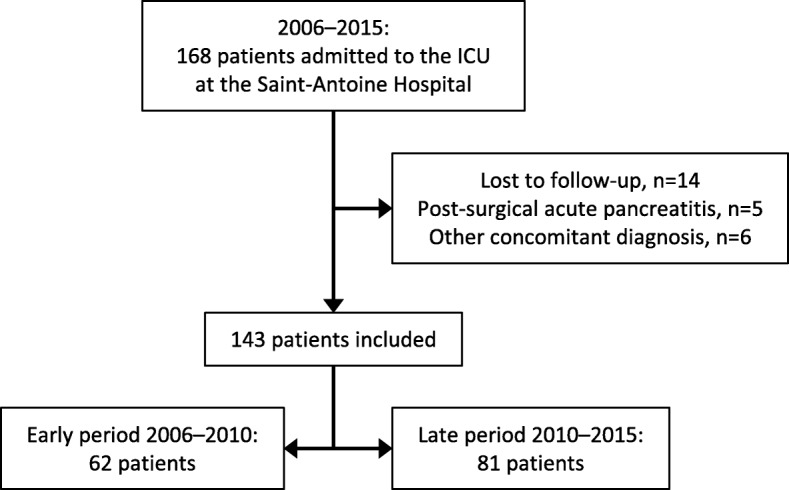
Study flowchart. Abbreviation: *ICU* intensive care unit

Figure 1 is the patient flowchart. Between September 2006 and August 2015, 143 patients were included: 62 before and 81 during and after January 2011. Table 1 reports their main features, which did not differ significantly between the two groups. IPN was documented in similar proportions of patients in the two groups.

**Table 1:** Main features of the study patients

	2006–2015 *n* = 143	2006–2010 *n* = 62	2011–2015 *n* = 81	*P* value
Patient characteristics				
Age in years, median [IQR]	59 [47–69]	58 [47–65]	60 [46–72]	0.3
Body mass index in kg/m^2^, median [IQR]	26 [23–29]	25 [22–29]	26 [24–29]	0.2
Males, n (%)	85 (59%)	40 (64%)	45 (55%)	0.9
Cause of pancreatitis, n (%)				
Alcoholism	46 (32%)	21 (34%)	25 (31%)	0.2
Lithiasis	60 (42%)	21 (34%)	39 (48%)	
Other	37 (26%)	20 (32%)	17 (21%)	
Balthazar score E, n (%)	116 (87.2%)	46 (82%)	70 (91%)	0.3
Extent of necrosis, n (%)				
None	31 (30%)	9 (24%)	22 (33%)	0.72
<30%	34 (32%)	16 (42%)	18 (27%)	
30–50%	10 (10%)	5 (13%)	5 (7%)	
>50%	30 (29%)	8 (21%)	22 (33%)	
SOFA score at ICU admission	4 [2–7]	4 [2–7]	4 [2–7]	0.9
Infected pancreatic necrosis, n (%)	80 (56%)	37 (60%)	43 (53%)	0.45
Lactate level in mmol/L, median [IQR]	1.9 [1.4–3.3]	2.2 [1.4–4.5]	1.9 [1.5–3.3]	0.6
Admission modality to our ICU, n (%)				
Admitted directly	92 (64%)	40 (65%)	52 (64%)	0.99
Transferred from another ICU	51 (36%)	22 (35%)	29 (36%)	

Abbreviations: *ICU* intensive care unit, *IQR* interquartile range, *SOFA* Sequential Organ Failure Assessment

### Management of infected pancreatic necrosis

**Fig. 2:**
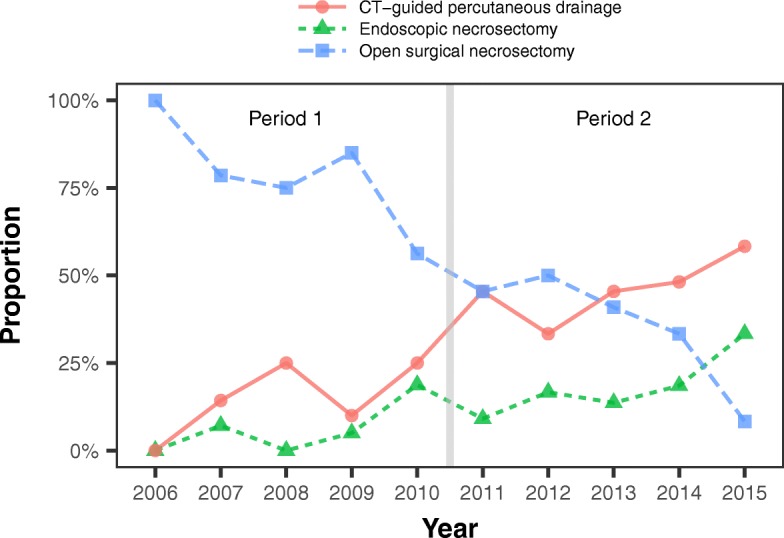
Change in drainage of infected pancreatic necrosis between the early (2006–2010) and late (2011–2015) periods. Abbreviation: *CT* computed tomography

The treatment strategies for suspected or proven IPN differed significantly between the groups. Overall, 75% of patients underwent drainage, and there was no difference between the early and late groups (81% versus 70%, respectively; *P* = 0.18). Figure 2 shows the changes over time in the use of OSN and of each of the two MID methods. Between 2010 and 2011, the proportion of patients managed with MID rose above the proportion managed with OSN. Thus, during the early period, OSN was used more often (74% versus 41%; *P* <0.001) and MID methods less often (19% versus 52%; *P* <0.001). More specifically, CT-guided percutaneous drainage was performed in 16% of patients in the early period versus 51% in the late period (*P* <0.0001), and endoscopic transgastric necrosectomy was performed in 8% and 20% of patients during these two periods, respectively (*P* = 0.059).

### Outcomes

**Fig. 3:**
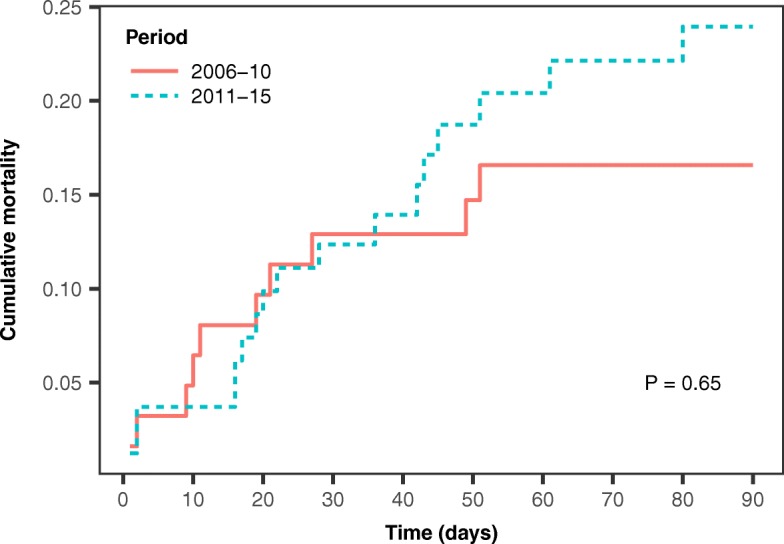
Ninety-day mortality rates

**Fig. 4:**
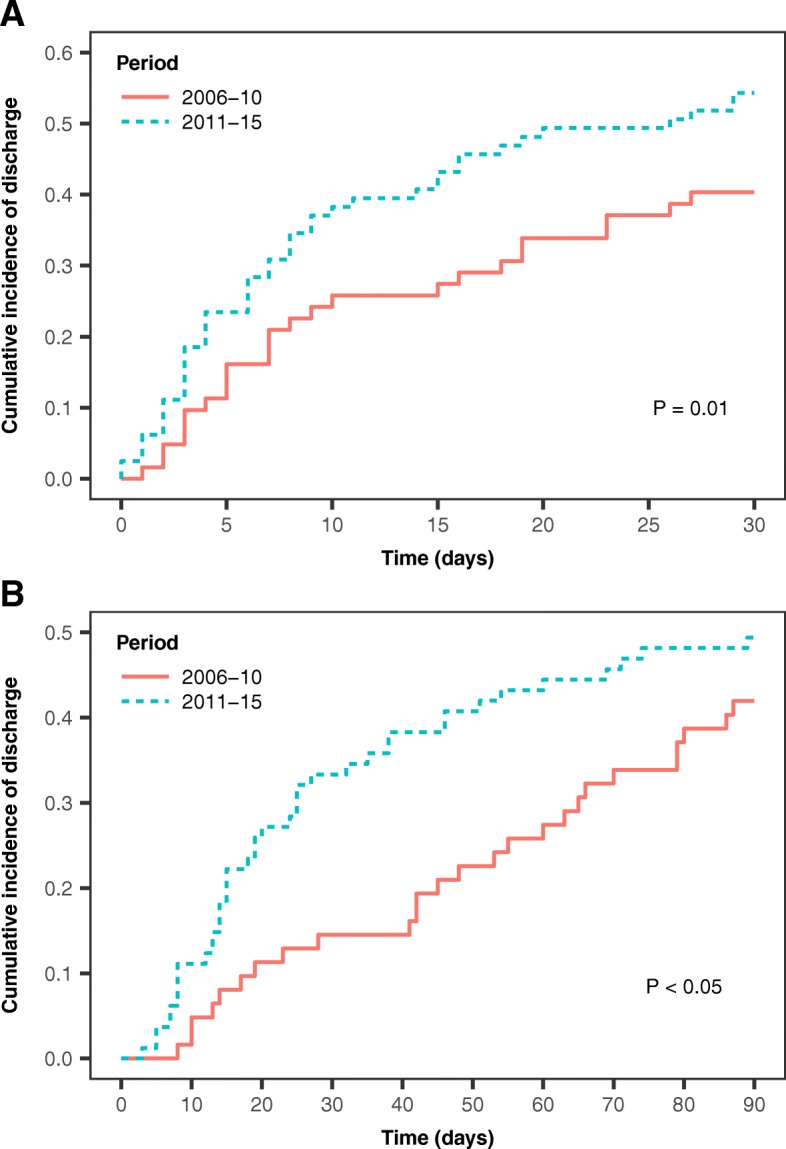
Time to discharge alive from the intensive care unit (**a**) and hospital (**b**) during the study periods: Gray estimator, with death as a competing risk

In-hospital mortality was 21% overall, and there was no significant difference between periods (early, 19%; late, 22%; *P* = 0.84). Ninety-day mortality, adjusted on age and baseline SOFA, was not significantly different (HR 1.20; 95% CI 0.11–12.75; *P* = 0.65) (Fig. 3). Both ICU and hospital stays were significantly longer during the early period: with death as a competing risk and after adjusting on age and baseline SOFA, patients in the late period were more likely to leave the ICU before day 30 (HR 1.93; 95% CI 1.17–3.19; *P* = 0.01) (Fig. 4a) and to leave the hospital before day 90 (HR 1.66; 95% CI 1.01–2.74; *P* <0.05) (Fig. 4b) than were patients in the early period.

**Fig. 5:**
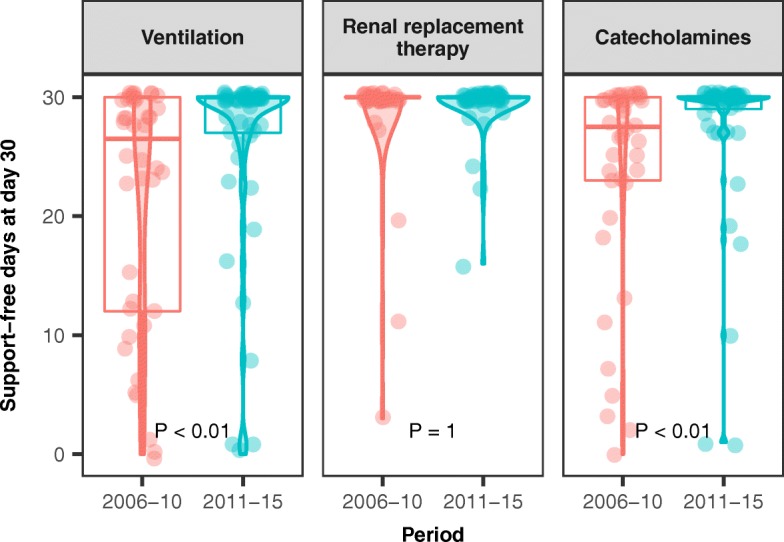
Organ dysfunction duration according to periods. Box-plots showing the numbers of days without mechanical ventilation, renal replacement therapy, and catecholamines during the first 30 days after intensive care unit admission. The horizontal lines, from top to bottom, are the 75th percentile, median, and 25th percentile

Complete data on organ support therapy were available for the 92 patients admitted directly to our ICU, as opposed to being transferred from another hospital; 40 were admitted during the early period and 51 during the late period. The duration of organ support therapy was shorter during the late period. The median number of MV-free days by day 30 was lower in the early period (26.5 [IQR 12–30] versus 30 [IQR 27–30] in the late period; *P* = 0.0015). The number of RRT-free days by day 30 was similar in the early and late groups, in which 5 (13%) and 7 (14%) patients, respectively, required dialysis or filtration therapy. The median number of catecholamine-free days by day 30 was lower in the early period (27.5 [IQR 23–30] versus 30 days [IQR 29–30]; *P* = 0.0015) (Fig. 5).

To assess the possibility that changes in other aspects of patient management over time may have introduced bias, we compared outcomes between the two periods in patients managed with MID and those managed with OSN. Ninety-day mortality adjusted on age and baseline SOFA was not significantly different between the two periods in the 54 patients managed with MID (12 during the early and 42 during the late period; HR 1.89; 95% CI 0.23–15.55; *P* = 0.55) or in the 79 patients managed with OSN (46 in the early and 33 in the late period; HR 1.83; 95% CI 0.70–4.77; *P* = 0.21). ICU and hospital stay lengths, with death as a competing risk and after adjustment on age and baseline SOFA, were not significantly different between the two periods in the MID group or in the OSN group. Thus, among patients managed with MID, those managed during the later period were as likely to leave the ICU before day 30 as were those managed during the early period (HR 1.99; 95% CI 0.62–6.43; *P* = 0.24). Similarly, hospital discharge before day 90 occurred in similar proportions of patients in the late period as in the early period (HR 0.75; 95% CI 0.27–2.05; *P* = 0.57). In the OSN group, results were similar for both ICU discharge before day 30 (HR 1.11; 95% CI 0.43–2.82; *P* = 0.83) and hospital discharge before day 90 (HR 0.97; 95% CI 0.37–2.52; *P* = 0.94).

## Discussion

In this study of a large cohort of critically ill patients with severe acute necrotizing pancreatitis, the use of MID instead of OSN as the first-line drainage method used to treat IPN was associated with shorter ICU and hospital stays and with shorter times on MV and hemodynamic support. There was no difference in 90-day in-hospital mortality.

These results are consistent with previous data from small randomized controlled trials in highly selected patients, most of whom were not critically ill. The Dutch PANTER randomized trial in 88 patients compared a surgical step-up approach and an endoscopic step-up approach involving percutaneous or transgastric endoscopic drainage followed, in the absence of improvement within 72 h, by retroperitoneal laparoscopy debridement [7]. The primary endpoint was a composite of death, multi-organ failure, perforation, and bleeding. This endpoint was reached significantly more often in the conventional surgery group than in the endoscopy group. However, overall mortality was not significantly different. Similarly, the substantially shorter ICU and hospital stays in our study after the change to first-line MID may reflect a decrease in digestive complications such as fistulae, bleeding, and perforation. These results support the hypothesis that the decreased systemic and local inflammation with MID compared with OSN expedites the control of infected necrotic lesions, obviating the need for early surgery (within 4 weeks), whose limitations have been demonstrated. Thus, in a study of 167 patients, surgery during the first 28 days was associated with significantly higher mortality (20%) compared with surgery performed later on (5%) [13]. MID techniques may induce less surgical trauma and a weaker inflammatory response compared with conventional surgery in patients who are already severely ill [14–16].

A significant finding from our study is the substantially shorter time on MV in the group managed chiefly using MID. Severe acute pancreatitis is often complicated by multiple organ system dysfunctions, notably pulmonary complications [17]. Overall mortality seems to correlate with the severity of hypoxemia when respiratory failure develops in patients with acute pancreatitis [18, 19]. There is growing evidence that pro-inflammatory cytokines such as interleukin-1 (IL-1), IL-6, and tumor necrosis factor-alpha (TNF-α) play a central role in the systemic complications of acute pancreatitis and may be involved in diaphragmatic dysfunction during sepsis [20–24]. In an experimental study in hamsters, high doses of TNF-α induced diaphragmatic dysfunction [25]. During acute pancreatitis, cytokine concentrations are elevated within the pancreas [22]. Furthermore, TNF-α and IL-1 may exert a synergistic negative inotropic effect on diaphragm contractility [26]. The PENGUIN study compared transgastric necrosectomy to OSN in 20 patients. The main endpoint was the inflammatory response to the procedure as assessed by the serum IL-6 levels [8]. The IL-6 level was significantly lower in the MID group. These data suggest that surgery-induced elevations in pro-inflammatory cytokines may promote diaphragmatic dysfunction, thereby increasing the length of MV.

Strengths of our study include the real-life setting and large number of patients. We chose to include patients transferred from other hospitals in order to reflect real-life practice. Our population was representative of patients with organ failure induced by severe pancreatitis. We thus obtained data on the sickest and most complex patients, which were lacking until now, as previous studies were not confined to ICU patients. The 10-year span of our retrospective study provides a clear picture of the changes in IPN management in our unit over time. The outcomes that were improved during the predominantly MID period have strong clinical implications. For instance, the shorter stay lengths and shorter MV times would be expected to translate into decreases in ICU-acquired complications such as ventilator-associated pneumonia. Major cost savings would also be expected given the high incidence of acute pancreatitis. In the US, 233,000 patients were admitted for acute pancreatitis in 2006 and about 5% of them developed IPN [27].

Our study also has limitations. While the findings of this study are consistent with the current view that MID techniques deserve preference, the before-after study design carries a risk of bias due to possible changes in other aspects of patient management over time. However, this potential bias was minimized by performing the study in a single specialized center. Furthermore, we compared the early and late periods regarding outcomes of patients managed with either MID or OSN. Ninety-day mortality and ICU and hospital lengths of stay did not differ between periods in either the MID or the OSN group. This finding supports the change in drainage strategy as the cause of the improvement in outcomes during the late compared with the early period. A caveat is in order, however, as MID may have been used preferentially in the sickest patients during the late period. However, these analyses provide the clearest possible picture of the independent impact of the change in drainage strategy.

Throughout the study period, access to interventional endoscopy techniques was still restricted and patients had to be transferred to another hospital for endoscopic necrosectomy, which explains the limited proportion of patients managed with this technique. Furthermore, owing to the retrospective design, clinical data were unavailable on some points such as the number of endoscopic necrosectomy procedures.

## Conclusions

In our study, the introduction of MID techniques to treat critically ill patients with IPN was associated with improvements in several clinical outcomes. Increasing the availability of MID techniques deserves to be viewed as a major therapeutic goal, especially for patients with the most severe forms of acute necrotizing pancreatitis.

## Abbreviations

CI: Confidence interval
CT: Computed tomography
HR: Hazard ratio
ICU: Intensive care unit
IL: Interleukin
IPN: Infected pancreatic necrosis
IQR: Interquartile range
MID: Minimally invasive drainage
MV: Mechanical ventilation
OSN: Open surgical necrosectomy
RRT: Renal replacement therapy
SOFA: Sequential Organ Failure Assessment
TNF-α: Tumor necrosis factor-alpha

## References

[1] Yadav D, Lowenfels AB (2013). The epidemiology of pancreatitis and pancreatic cancer. Gastroenterology.

[2] Banks PA, Freeman ML (2006). Practice guidelines in acute pancreatitis. Am J Gastroenterol.

[3] Tenner S, Sica G, Hughes M, Noordhoek E, Feng S, Zinner M (1997). Relationship of necrosis to organ failure in severe acute pancreatitis. Gastroenterology.

[4] Buter A, Imrie CW, Carter CR, Evans S, McKay CJ (2002). Dynamic nature of early organ dysfunction determines outcome in acute pancreatitis. Br J Surg.

[5] Beger HG, Rau B, Mayer J, Pralle U (1997). Natural course of acute pancreatitis. World J Surg.

[6] De Waele JJ, Hoste E, Blot SI, Hesse U, Pattyn P, de Hemptinne B (2004). Perioperative factors determine outcome after surgery for severe acute pancreatitis. Crit Care.

[7] van Santvoort HC, Besselink MG, Bakker OJ, Hofker HS, Boermeester MA, Dejong CH (2010). A Step-up Approach or Open Necrosectomy for Necrotizing Pancreatitis. N Engl J Med.

[8] Bakker OJ, van Santvoort HC, van Brunschot S, Geskus RB, Besselink MG, Bollen TL (2012). Endoscopic transgastric vs surgical necrosectomy for infected necrotizing pancreatitis: a randomized trial. JAMA.

[9] van Brunschot S (2018). Endoscopic or surgical step-up approach for necrotizing pancreatitis, a multi-centre randomized controlled trial. Lancet.

[10] Van Baal MC, Van Santvoort HC, Bollen TL, Bakker OJ, Besselink MG, Gooszen HG (2011). Systematic review of percutaneous catheter drainage as primary treatment for necrotizing pancreatitis. British J Surg.

[11] Paye F, Frileux P, Lehman P, Ollivier JM, Vaillant JC, Parc R (1999). Reoperation for severe pancreatitis: a 10-year experience in a tertiary care center. Arch Surg.

[12] Gray RJ (1988). A Class of K-Sample Tests for Comparing the Cumulative Incidence of a Competing Risk. Ann Stat Inst Math Stat.

[13] Rodriguez JR, Razo AO, Targarona J, Thayer SP, Rattner DW, Warshaw AL (2008). Debridement and closed packing for sterile or infected necrotizing pancreatitis: Insights into indications and outcomes in 167 patients. Ann Surg.

[14] Connor S, Alexakis N, MGT R, Ghaneh P, Evans J, Hughes M (2005). Early and late complications after pancreatic necrosectomy. Surg.

[15] Carter CR, McKay CJ, Imrie CW (2000). Percutaneous necrosectomy and sinus tract endoscopy in the management of infected pancreatic necrosis: an initial experience. Ann Surg.

[16] van Santvoort HC, Besselink MGH, Horvath KD, Sinanan MN, Bollen TL, van Ramshorst B (2007). Videoscopic assisted retroperitoneal debridement in infected necrotizing pancreatitis. HPB.

[17] Browne GW, Pitchumoni CS (2006). Pathophysiology of pulmonary complications of acute pancreatitis. World J Gastroenterol.

[18] Imrie CW, Ferguson JC, Murphy D, Blumgart LH (1977). Arterial hypoxia in acute pancreatitis. Br J Surg.

[19] Murphy D, Imrie CW, Pack A, Davidson JF, Blumgart LH (1976). Proceedings: The mechanism of acute respiratory insufficiency in acute pancreatitis. Br J Surg.

[20] Heath DI, Cruickshank A, Gudgeon M, Jehanli A, Shenkin A, Imrie CW (1993). Role of interleukin-6 in mediating the acute phase protein response and potential as an early means of severity assessment in acute pancreatitis. Gut.

[21] Matuszczak Y, Viirés N, Aubier M, Desmonts JM, Dureuil B (1998). Diaphragmatic function is markedly altered in cerulein-induced pancreatitis. Crit Care Med.

[22] Norman JG, Fink GW, Denham W, Yang J, Carter G, Sexton C (1997). Tissue-specific cytokine production during experimental acute pancreatitis: a probable mechanism for distant organ dysfunction. Dig Dis Sci.

[23] Norman JG, Franz MG, Fink GS, Messina J, Fabri PJ, Gower WR (1995). Decreased mortality of severe acute pancreatitis after proximal cytokine blockade. Ann Surg.

[24] Nieminen A, Maksimow M, Mentula P, Kyhälä L, Kylänpää L, Puolakkainen P (2014). Circulating cytokines in predicting development of severe acute pancreatitis. Crit Care.

[25] Wilcox P, Milliken C, Bressler B (1996). High-dose Tumor Necrosis Factor a Produces an Impairment of Hamster Diaphragm Contractility: Attenuation with a Prostaglandin Inhibitor. Am J Respir Crit Care Med.

[26] Wilcox P, Osborne S, Bressler B (1992). Monocyte inflammatory mediators impair in vitro hamster diaphragm contractility. Am Rev Respir Dis.

[27] DeFrances CJ, Lucas CA, Buie VC, Golosinskiy A (2008). 2006 National Hospital Discharge Survey. Natl Heal Stat Rep.

